# Uncover New Reactivity of Genetically Encoded Alkyl Bromide Non-Canonical Amino Acids

**DOI:** 10.3389/fchem.2022.815991

**Published:** 2022-02-18

**Authors:** Xin Shu, Sana Asghar, Fan Yang, Shang-Tong Li, Haifan Wu, Bing Yang

**Affiliations:** ^1^ Zhejiang Provincial Key Laboratory for Cancer Molecular Cell Biology, Life Sciences Institute, Zhejiang University, Hangzhou, China; ^2^ Cancer Center, Zhejiang University, Hangzhou, China; ^3^ Department of Biophysics, Kidney Disease Center of the First Affiliated Hospital, Zhejiang University School of Medicine, Hangzhou, China; ^4^ Glbizzia Biosciences Co., Ltd, Beijing, China; ^5^ Department of Chemistry and Biochemistry, Wichita State University, Wichita, KS, United States

**Keywords:** protein-protein interactions, genetic code expansion, non-canonical amino acid, chemical cross-linking, and SUMO interactome

## Abstract

Genetically encoded non-canonical amino acids (ncAAs) with electrophilic moieties are excellent tools to investigate protein-protein interactions (PPIs) both *in vitro* and *in vivo*. These ncAAs, including a series of alkyl bromide-based ncAAs, mainly target cysteine residues to form protein-protein cross-links. Although some reactivities towards lysine and tyrosine residues have been reported, a comprehensive understanding of their reactivity towards a broad range of nucleophilic amino acids is lacking. Here we used a recently developed OpenUaa search engine to perform an in-depth analysis of mass spec data generated for Thioredoxin and its direct binding proteins cross-linked with an alkyl bromide-based ncAA, BprY. The analysis showed that, besides cysteine residues, BprY also targeted a broad range of nucleophilic amino acids. We validated this broad reactivity of BprY with Affibody/Z protein complex. We then successfully applied BprY to map a binding interface between SUMO2 and SUMO-interacting motifs (SIMs). BprY was further applied to probe SUMO2 interaction partners. We identified 264 SUMO2 binders, including several validated SUMO2 binders and many new binders. Our data demonstrated that BprY can be effectively used to probe protein-protein interaction interfaces even without cysteine residues, which will greatly expand the power of BprY in studying PPIs.

## Introduction

Protein-protein interactions (PPIs) are essential for virtually all cellular processes in all living organisms. Thus, there is a significant effort in mapping protein-protein interaction networks to understand relevant biological processes in detail, and many techniques have been developed for this purpose (Low et al., 2021). Affinity purification mass spectrometry (AP-MS) have been successfully applied to map protein-protein interactomes in many organisms (Ho et al., 2002; Butland et al., 2005; Krogan et al., 2006; Gordon et al., 2020; Richards et al., 2021), although these methods cannot distinguish between direct binders and indirect binders of a protein of interest (POI) (Morris et al., 2014). Moreover, weak and transient interactions are typically not comprehensively detected under the conditions of AP-MS (Richards et al., 2021). Proximity labeling (PL) by introducing covalent labels to proteins proximal to a POI allows large scale analysis of protein-protein interactions with potential spatial and temporal resolutions in cells (Han et al., 2018; Roux et al., 2018). However, limited information is available to map the interaction interfaces to gain further structural understanding of these interactions.

**FIGURE 1:**
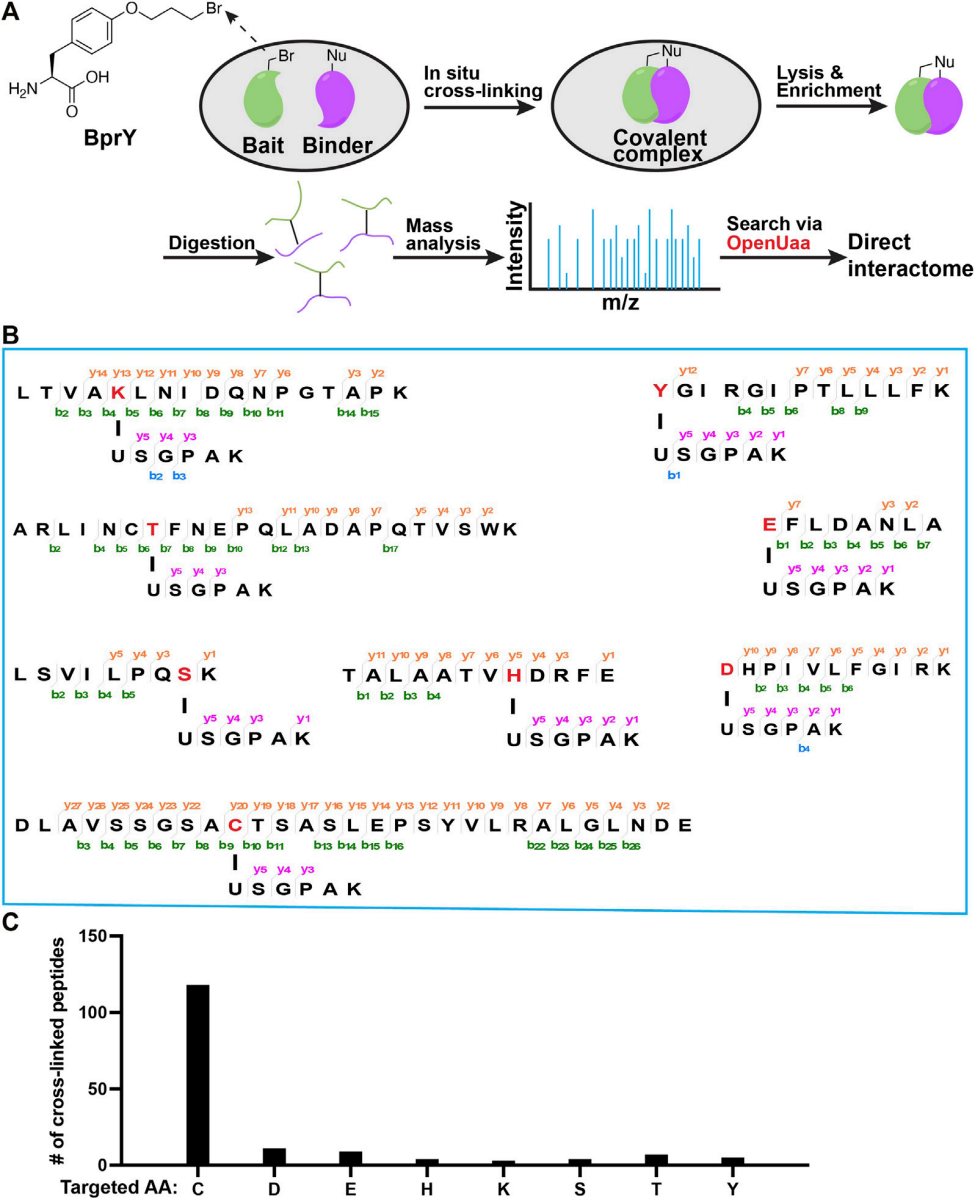
Broad reactivity of BprY to nucleophilic amino acids identified by data mining. **(A)** Scheme of *in-situ* BprY cross-linking and in-depth data analysis by OpenUaa. **(B)** MS/MS fragmentation patterns of cross-linked peptides with different nucleophilic AAs targeted by BprY. **(C)** Number of cross-linked peptides identified for different targeted AAs.

A complementary method to AP-MS and PL for analyzing PPIs involves covalent cross-linking (Liu et al., 2015; Wang, 2017; Nguyen et al., 2018; Yu and Huang, 2018). Genetic code expansion by amber codon suppression has enabled site-specific incorporation of non-canonical amino acids (ncAAs) into proteins (Wang et al., 2001; Wang and Schultz, 2004; Wang et al., 2006; de la Torre and Chin, 2021; Shandell et al., 2021). Many ncAAs, including photo-activated ncAAs (Chin et al., 2002a; Chin et al., 2002b; Zhang et al., 2011; Lin et al., 2014; Yang et al., 2016) and those with fine-tuned bio-reactivity to capture protein binders (Xiang et al., 2013; Chen X.-H. et al., 2014; Furman et al., 2014; Xiang et al., 2014; Xuan et al., 2016; Cigler et al., 2017; Wang, 2017; Nguyen et al., 2018; Shang et al., 2018; Wang et al., 2018; Yang et al., 2019; Liu et al., 2020; Liu J. et al., 2021), has been successfully incorporated into proteins. One example is a chemical cross-linking ncAA BprY with an electrophilic alkyl bromide group (Figure 1A), which is typically unreactive towards biomolecules in cells after incorporation into a POI unless there is a proximal nucleophilic cysteine residue from the binder of this POI through the proximity-enabled reactivity. Given its ability to cross-link proximal cysteine residues, BprY has been successfully applied to capture proteome-wide protein-protein interactions in live cells (Yang et al., 2017), allowing the development of GECX-MS (Genetically Encoded Chemical Cross-linking of proteins coupled with Mass Spectrometry) as a powerful tool to identify direct binding partners of target proteins (Figure 1A). In GECX-MS, a cross-linkable ncAA, such as BprY, is genetically incorporated into a bait protein to covalently capture binder proteins including those weak and transient PPIs *in situ*. Analysis of cross-linked peptides by mass spectroscopy identifies not only binder proteins but also their corresponding cross-linking sites, which could be used to map binding interfaces or binding motifs.

One limitation of BprY and related alkyl halide-based ncAAs is that the cross-linking reaction requires proximal cysteine residues in binder proteins. Because cysteine residues are in relatively low abundance and they often form disulfide bonds, potentially binder proteins without proximal cysteine residues may not be captured. To maximize the potential of BprY to probe protein-protein interactions, its amino acid reactivity beyond cysteine is critical. Although there are some reports of extended reactivity of alkyl bromide based ncAAs towards glutamate, lysine and histidine (Chen X.-H. et al., 2014; Xiang et al., 2014; Cigler et al., 2017), a comprehensive analysis of BprY reactivity towards different nucleophilic amino acids under physiological conditions is not available. Here we show that BprY can target a broad range of nucleophilic amino acid residues, including Cys, Asp, Glu, Ser, Thr, His, and Tyr, among which the reactivity to Cys is still the highest. BprY can also be used to probe PPIs without cysteine at the binding interface. Finally, we successfully applied BprY to identify SUMO2-interacting proteins at a whole proteome level.

## Materials and Methods

### Plasmid Construction

*pBad-Affibody and mutants*. The gene encoding Affibody was PCR amplified with Affibody-NdeI-F and Affibody-HindIII-R primers, and the PCR product was cloned into a commercial pBad vector pre-treated with NdeI and HindIII enzymes. Affibody-D2N mutant was generated by PCR amplification of pBad-Affibody with Affibody-D2N-F and Affibody-HindIII-R primers. Affibody mutants pBad-Afb*(K7X) were generated by PCR amplification of pBad-Affibody with Affibody-Mutant-F and Affibody-Mutant-R primers.

Affibody-NdeI-F:

GGA​GAT​ATA​CAT​ATG​GTA​GAC​AAC​GCC​TTC​AAC

Affibody-HindIII-R:

AAA​ACA​GCC​AAG​CTT​TTA​GTG​ATG​GTG​ATG​GTG​ATG​A

Affibody-D2N-F:

GGA​GAT​ATA​CAT​ATG​GTA​AAC​AAC​GCC​TTC​AAC​AAG

Affibody-Mutant-F:

AACAACGCCTTCAACxxxCAACTATCAGTCGCC

Affibody-Mutant-R:

GGCGACTGATAGTTGxxxGTTGAAGGCGTTGTT

*pBad-MBP-Z-24TAG*. The gene encoding MBP-Z fusion protein was PCR amplified with the following primers, and the PCR product was cloned into the pBad vector pre-digested with NdeI and HindIII enzymes. pBad-MBP-Z-24TAG was generated by PCR amplification of pBad-MBP-Z with MBP-Z-24TAG-E25Q-Mutant primers.

MBP-Z-NdeI-F:

GGA​GAT​ATA​CAT​ATG​ATG​AAA​ATC​GAA​GAA​GGT​AAA​CTG

MBP-Z-HindIII-R:

CAA​AAC​AGC​CAA​GCT​TTT​AAT​GAT​GAT​GAT​GAT​GAT​GCT​TAG​G

MBP-Z-24TAG-E25Q-Mutant-F:

TTA​CCT​AAC​CTG​AAT​TAG​CAG​CAG​CGT​AAT​GCC​TTC

MBP-Z-24TAG-E25Q -Mutant-R:

GAA​GGC​ATT​ACG​CTG​CTG​CTA​ATT​CAG​GTT​AGG​TAA

*pBad-SUMO2 and mutants*. The gene encoding *Homo sapiens* SUMO-2 (NCBI Reference Sequence: NM_006937.3) was PCR amplified with the following primers, and the PCR product was cloned into the pBad vector pre-treated with NdeI and HindIII enzymes. Sites of hSUMO2-E49 and R50 were mutated to a TAG codon respectively.

hSUMO2-NdeI-F:

GGA​GAT​ATA​CAT​ATG​ATG​GCC​GAC​GAA​AAG​C

hSUMO2-HindIII-R:

AAC​AGC​CAA​GCT​TTC​AGT​GAT​GGT​GAT​GGT​GAT​GGT​AGA​CAC​CTC​CCG​TCT

*pET28a-MBP-RNF111*_*293-391*_. The gene encoding *Homo sapiens* RNF111_293-391_ (NCBI Reference Sequence: NM_001270530.1) was PCR amplified with the following primers, and the PCR product was cloned into a pET28a-MBP vector.

pET28a-MBP-RNF111-F:

AAG​TTC​TGT​TCC​AGG​GGC​CCC​ATA​TGA​TGT​CAG​GAA​GTA​TTG​ATG​AAG​ATG​TTG

pET28a-MBP-RNF111-R:

CAG​TGG​TGG​TGG​TGG​TGG​TGC​TCG​AGT​TCA​TCT​TCA​TCA​ACG​GTA​AGG​TC

### Protein Expression

*Affibody, SUMO2 and MBP-RNF111*_*293-391*_. The corresponding plasmids were individually transformed into DH10B cells, which were plated on LB agar plates supplemented with 100 μg/mL ampicillin. Colonies were picked from the plate of each plasmid and individually inoculated to 100 mL LB (5 g/L NaCl, 10 g/L Tryptone, 10 g/L Yeast extract). Cells were grown at 37°C and 200 rpm to an OD of 0.6 with good aeration and the relevant antibiotic selection. For induction of MBP-RNF111_293-391_, 400 μM IPTG was added. 0.2% L-arabinose was added to induce the expression of Affibody and SUMO2. The expression was carried out at 30°C, 200 rpm for 5h. Cells were harvested by centrifugation at 6,000 *g*, 4°C for 10 min. The cell pellet was washed with cold PBS buffer and centrifuged again at 6,000 g, 4°C for 10 min. Cell pellets were then frozen in liquid nitrogen and stored at −80°C.

ncAA constructs: MBP-Z(E24BprY), SUMO2*(E49BprY), and SUMO2*(R50BprY). The corresponding plasmids were individually transformed into DH10B cells together with pEvol-BprY plasmid. Cells were plated on LB agar plates supplemented with 100 μg/mL ampicillin and 30 μg/mL chloramphenicol. Colonies were picked from these plates and individually inoculated to 25 mL LB (5 g/L NaCI, 10 g/L Tryptone, 10 g/L Yeast extract). Cells were grown at 37°C and 200 rpm to an OD of 0.4 with good aeration and the relevant antibiotic selection. Then the medium was added 1 mM ncAA BprY and 0.2% L-arabinose. The expression was carried out at 18°C and 200 rpm shaking for 18 h. Cells were harvested by centrifugation at 3,260 *g* and 4°C for 20 min. Cell pellets were washed with cold PBS buffer, centrifuged again at 3,260 g and 4°C for 20 min. Cell pellets were then frozen in liquid nitrogen and stored at −80°C.

### His-Tag Protein Purification

Frozen cells were rapidly thawed and resuspended in lysis buffer (50mM Tris, pH8.0, 500 mM NaCl, 0.1% Tween-20). EDTA S3 free protease inhibitor cocktail was added followed by vortexing for 2 min. Cells were lysed by sonication after which the cell lysate was clarified by centrifugation at 13,000 rpm and 4°C for 30 min. The supernatant was collected and incubated with 200 µL Ni-NTA Affinity resin at 4°C for 1 h. The resin was washed with an equal volume of wash buffer (50 mM Tris pH8.0, 500 mM NaCl, 20 mM imidazole) for 2 times at 4°C. Elution was done with 200 µl elution buffer (50 mM Tris pH8.0, 100 mM NaCl, 250 mM imidazole) for five times. The fractions containing the target protein were determined by SDS-PAGE analysis with 10% Tricine gel.

### Proteins Cross-Linking *in vitro*

A 20 μL reaction mixture containing MBP-Z(E24BprY) (0.6 mg/mL) and Affibody (1.2 mg/mL) in HEPES buffer (pH 7.5) was incubated at 37°C for 8 h. A 20 μL reaction mixture containing SUMO2 (10 μM) and RNF111 (293-391) (10 μM) in HEPES buffer (pH 7.5) was incubated at 37°C for 8 h.

### Protein Digestion

Protein samples were precipitated by the addition of six volumes of cold acetone (−20°C) and incubated at −20°C for 30 min. Precipitated proteins were air dried and resuspended in 8 M urea, 100 mM Tris, pH 8.5. After reduction with 5 mM TCEP for 20 min and alkylation with 10 mM iodoacetamide for 15 min in the dark, samples were diluted to 2 M urea with 100 mM Tris, pH 8.5, and digested with trypsin (at 50:1 protein: enzyme ratio) at 37°C for 16 h. Digestion was terminated by adding formic acid to 5% final concentration, and digested peptides were desalted with StageTips.

### Cell Lysate Cross-Linking and Two-step His-Tag Purification to Enrich Cross-Linked Peptides

Cell pellets expressing His-tagged SUMO2*(E49BprY) or SUMO2*(R50BprY) were resuspended in 4 ml lysis buffer (50 mM Tris, pH 8.0, 500 mM NaCl, 0.1% Tween-20), separately. Cells were lysed by sonication after which the cell lysate was clarified by centrifugation at 13,000 rpm and 4°C for 30 min. The supernatants were collected and incubated with 200 µl Ni-NTA Affinity resin at 4°C for 2 h. The resin was washed with 4 ml of wash buffer (50 mM Tris pH 8.0, 500 mM NaCl, and 20 mM imidazole) for 3 times at 4°C, followed by a second rinsed with an equal volume of wash buffer 2 (50 mM Tris pH8.0, 500 mM NaCl, 40 mM imidazole) for 3 times at 4°C. Then the resin was equilibrated with lysis buffer and incubated with 293T cell lysates from one 10 cm plate at RT for overnight. The next day, the resin was washed with 4 mL wash buffer (50 mM Tris pH 8.0, 500 mM NaCl, 20 mM imidazole) for 3 times at 4°C, and then rinsed with an equal volume of wash buffer 2 (50 mM Tris pH8.0, 500 mM NaCl, 40 mM imidazole) for 3 times at 4°C. Elution was done with 200 µl elution buffer (50 mM Tris pH 8.0, 100 mM NaCl, 250 mM imidazole) for five times. The eluates were concentrated, and buffer exchanged into 100 µl of protein storage buffer (50 mM HEPES, pH 7.5, and 100 mM NaCl) using 10 k Amicon Ultra columns. Purified proteins were digested with Lys-C at 37°C for overnight, and digested peptides were incubated with pre-equilibrated Ni-NTA Agarose resin (50 µL) at 4°C for 2 h to further enrich cross-linked peptides (all contain C-terminal His tag after Lys-C digestion). Resin was rinsed with wash buffer 2 for three times and 50 mM NH_4_HCO_3_ twice. Bound peptides were digested on-bead with Trypsin at 37°C for 8 h, and digested peptides were desalted with StageTips before MS analysis.

### Tandem MS Analysis

MS experiments were performed on a Q Exactive HF-X instrument (ThermoFisher) coupled with an Easy-nLC 1200 system. Mobile phase A and B were water and 80% acetonitrile, respectively, with 0.1% formic acid. Digested peptides were loaded directly onto analytical column (75 μm × 20 cm, 1.9 μm C18, 5 μm tip) at a flow rate of 300 nL/min. All peptide samples were separated using a linear gradient of 6–22% B for 38 min, 22–35% B for 17 min, 35–90% B in 2 min, 90% B for 1 min, 100% B for 2 min. Survey scans of peptide precursors were performed from 350 to 1500 *m*/*z* at 60,000 FWHM resolution with a 1 × 10^6^ ion count target and a maximum injection time of 20 ms. The instrument was set to run in top-speed mode with 1-s cycles for the survey and the MS/MS scans. After a survey scan, tandem MS was then performed on the most abundant precursors exhibiting a charge state from 3 to 7 of greater than 1 × 10^5^ intensity by isolating them in the quadrupole at 1.6 m/z. Higher energy collisional dissociation (HCD) fragmentation was applied with 27% collision energy and resulting fragments detected in the Orbitrap detector at a resolution of 15,000. The maximum injection time limited was 30 ms, and dynamic exclusion was set to 30 s with a 10 ppm mass tolerance around the precursor.

### MS Data Analysis

MS/MS spectra were extracted by parsing from RAW file. Datasets of model proteins were searched against the corresponding proteins by OpenUaa. OpenUaa was also used to search data of two-step purified Trx sample and SUMO interaction protein sample against *E. coli* proteome and human proteome downloaded from the UniProt database and the reversed decoy proteins, separately. OpenUAA search parameters: 5% false discovery rate (FDR) at the peptide-spectrum match (PSM) level, 10 ppm precursor mass tolerance, 20 ppm fragment mass tolerance, variable modification Cys 57.02146, and three maximum number of missed cleavage sites.

### Molecular Docking Study

For molecular docking, we first relaxed the structures of SUMO2 and RNF4 (PDB ID: 6JXX and 4PPE, respectively) in the Rosetta software suite (Leaver-Fay et al., 2011). 1000 models were generated for each protein and the model with the lowest total score was chosen for docking. In the protein-protein docking using Rosetta suite, RNF4 was first randomly orientated relative to SUMO2, and then 10,000 docking models were generated. The final docking model was chosen as the model with the largest binding energy (Rosetta energy term: I_sc). The docking model was rendered in the UCSF Chimera (Pettersen et al., 2004).

### Bioinformatic Analysis

For the analysis of sequence motif around the cross-linked sites, MEME software was used to scan a ±15 amino acids sequence window around the cross-linked site to generate a consensus motif. Gene ontology (GO) term and KEGG pathway enrichment for functional analysis were performed using the clusterProfiler package under the R software (Yu et al., 2012). The human proteome was used as the background for enrichment analysis. The significance of the enrichment analysis was defined using a hypergeometric test, and the resulted *p* values were corrected for multiple hypothesis testing using the BH method (Hendriks and Vertegaal, 2016). The final reported enriched terms and pathways were filtered according to the adjusted *p* values <0.05. STRING network analysis was performed using the STRING database, which integrate multiple information sources for protein interaction speculation (Szklarczyk et al., 2019). Using all identified interacting proteins of SUMO2 as input, protein interactions between SUMO2 interacting proteins were filtered at a STRING interaction confidence of >0.4. Statistical analysis of the interaction network was performed based on the interactions in the networks compared to randomly expected frequency of interactions. Network visualization was performed using Cytoscape software version 3.7.2. The MCODE plugin of Cytoscape was used to extract the 8 interconnected modules under default settings for other parameters (Bader and Hogue, 2003).

## Results

*Deep mining of interactome data identified a broad reactivity of BprY*. We chose to investigate those nucleophilic amino acids known to carry out alkylation reactions (deGruyter et al., 2017). Nucleophilic substitution reactions with Asn/Gln/Arg sidechains are rare. Thus, we think they are unlikely to react with BprY. For Met, alkylation with benzyl bromides has been reported using model peptides under acidic conditions (Kramer and Deming, 2013). It is also known that iodoacetamide, a common alkylation reagent used in proteomics, can alkylate Met (Kruger et al., 2005). Therefore, we think it is possible that Met might also react with BprY. One concern is that the alkylation product sulfonium can undergo a dealkylation reaction (Kramer and Deming, 2013) and can dissociate upon collision to cause neutral mass loss (Kruger et al., 2005), which can further complicate the MS analysis. Due to these reasons, we decided to focus on Cys, Asp, Glu, His, Lys, Ser, Thr and Tyr in the current study. To systematically evaluate the reactivity of BprY to different types of amino acid residues in proteins, we re-analyzed previously published direct interactome data (Yang et al., 2017) of thioredoxin (Trx) probed by BprY (Figure 1A). Using a recently developed searching algorism OpenUaa (Liu C. et al., 2021), which allowed deeper mining of the interactome data, we observed a broad reactivity of BprY to multiple nucleophilic amino acids—Cys, Asp, Glu, His, Lys, Ser, Thr and Tyr, supported by high-quality MS/MS spectra (Figures 1B; Supplementary Figure S1). As expected, the reactivity is dominated by Cys with 118 Cys-targeted peptides identified. However, we also observed 43 non-Cys cross-linked peptides which still made a significant contribution to determining the direct interactome (Figure 1C).

**FIGURE 2:**
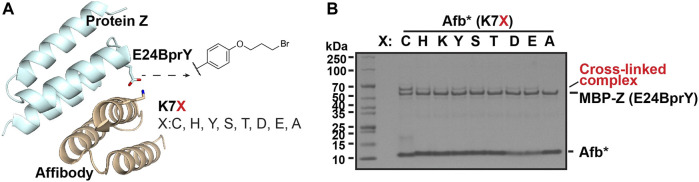
Broad reactivity of BprY to nucleophilic AAs demonstrated using a model interacting protein pair. **(A)** The interaction between protein Z and affibody (PDB ID 1LP1) shows proximity between residue E24 in protein Z and residue K7 in affibody. **(B)** Cross-linking between MBP-Z(E24BprY) to Afb*(K7X). * denotes Afb with triple mutants D2N, K4A, and E8Q.

*Validate the reactivity using a model interacting protein pair*. To validate this broad reactivity of BprY, we employed a model interacting protein pair—affibody (Afb) and protein Z fused to maltose-binding protein (MBP-Z) (Figure 2A). Efficient cross-linking was observed when a Cys residue and BprY were introduced to replace residue K7 of Afb and residue E24 of MBP-Z, respectively (Supplementary Figure S2A), consistent with a previous study (Yang et al., 2017). Several other AAs were also introduced at residue 7 of Afb, and the extent of cross-linking between Afb(K7X, X: variable AA) and MBP-Z(E24BprY) was evaluated. To our surprise, even some degree of cross-linking was observed between the “unreactive” control Afb(K7A) and MBP-Z(E24BprY) (Supplementary Figure S2A), suggesting nucleophilic amino acid residues adjacent to residue 7 in Afb can also facilitate cross-linking. We found that a triple mutation D2N, K4A, and E8Q of Afb completely eliminated cross-linking of the “unreactive” control (Figure 2B). Therefore, this triple mutation of Afb, denoted as Afb* thereafter, was further investigated. As shown in Figure 2B, Afb*(K7C) was efficiently cross-linked to MBP-Z(E24BprY), suggesting that the triple mutation didn’t affect the binding of Afb to MBP-Z. Further cross-linking of MBP-Z with Afb*(K7X) showed that, compared to Afb*(K7C), several nucleophilic amino acids displayed reactivity to BprY, although the cross-linking efficiency as evaluated from the intensity of the cross-linking band is lower than that of Cys.

**FIGURE 3:**
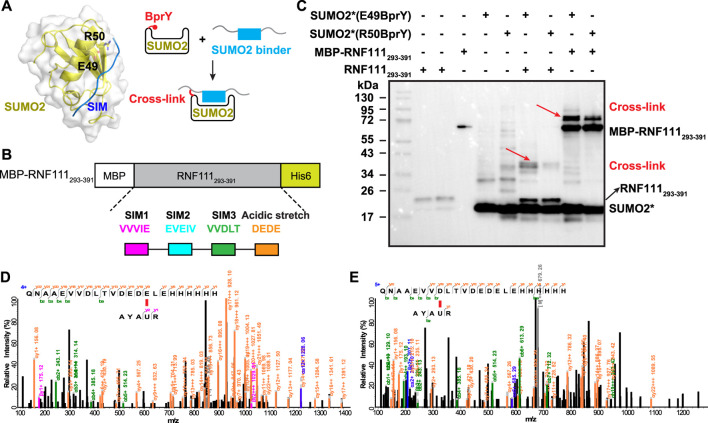
BprY successfully probed a PPI interface without Cys. **(A)** The interaction between SUMO2 and SIM (PDB ID 2MP2) shows residues E49 and R50 next to the SIM-binding groove. A scheme illustrating the incorporation of BprY at residue 49 or 50 of SUMO2 to covalently capture SUMO2 binding proteins. **(B)** The construct of MBP-RNF111_293-391_. **(C)** SDS-PAGE gel shows cross-linking of MBP-RNF111_293-391_ and RNF111_293-391_ with SUMO2* (E49BprY) or SUMO2* (R50BprY). Gel was stained by Coomassie brilliant blue. * denotes C48A mutation. Red arrows indicate cross-linking bands. **(D,E)** Representative MS/MS spectra of cross-linked peptides showing BprY in SUMO2 cross-linked to E391 and D384 of RNF111.

*Apply BprY to study PPI without Cys at the interface in vitro*. The study with model interacting protein pair further supported that BprY can target multiple nucleophilic amino acid residues beyond Cys, suggesting that BprY can be used to probe protein-protein interactions even without Cys residues at the interaction interface. To test this idea, we used BprY to capture the interaction between small ubiquitin-like modifiers (SUMOs) and one of their binding partners—RNF111. SUMOs can be reversibly conjugated to lysine side-chains of target proteins by an enzymatic cascade involving E1-E2-E3 enzymes, and this modification plays a key role in genome stability and transcription. The interaction between SUMOs and their binding partners is mediated primarily by SUMO-interacting motifs (SIMs) containing 3-4 aliphatic amino acid residues (Figure 3A) (Flotho and Melchior, 2013). RNF111 is a SUMO-targeted ubiquitin ligase with three SIMs and an acidic stretch adjacent to SIM3 (Poulsen et al., 2013; Sriramachandran et al., 2019).

To probe the binding between SUMO2 and RNF111, we generated two SUMO2 constructs with BprY individually incorporated into SUMO2 at residue E49 or R50, adjacent to the SIM-binding groove (Figure 3A). In both constructs, residue C48 was mutated to Ala to prevent intra-protein cross-linking. Mutation of these residues has been known to have a small effect on SUMO2 binding (Bouchenna et al., 2019; Bruninghoff et al., 2020). These two constructs SUMO2*(E49BprY) and SUMO2*(R50BprY) were then individually incubated with MBP-RNF111_293-391_ (Figure 3B). As shown in Figure 3C, both SUMO2*(E49BprY) and SUMO2*(R50BprY) formed inter-protein cross-links with MBP-RNF111_293-391_. The same result was also observed with RNF111_293-391_, suggesting the cross-links are specific to RNF111. Incorporation of BprY at residue 49 of SUMO2* appeared to give more efficient cross-linking, suggesting that the side-chain of residue 49 might be better positioned for the cross-linking reaction. Another possibility is that mutation of R50 caused weaker binding to SIMs. Further mass analysis identified cross-links between BprY in SUMO2* to residues D384 in SIM3 and E391 in the acidic stretch of RNF111 (Figures 3D,E), supporting that this acidic stretch contributes to the binding of SIM3 in RNF111 to SUMO2.

**FIGURE 4:**
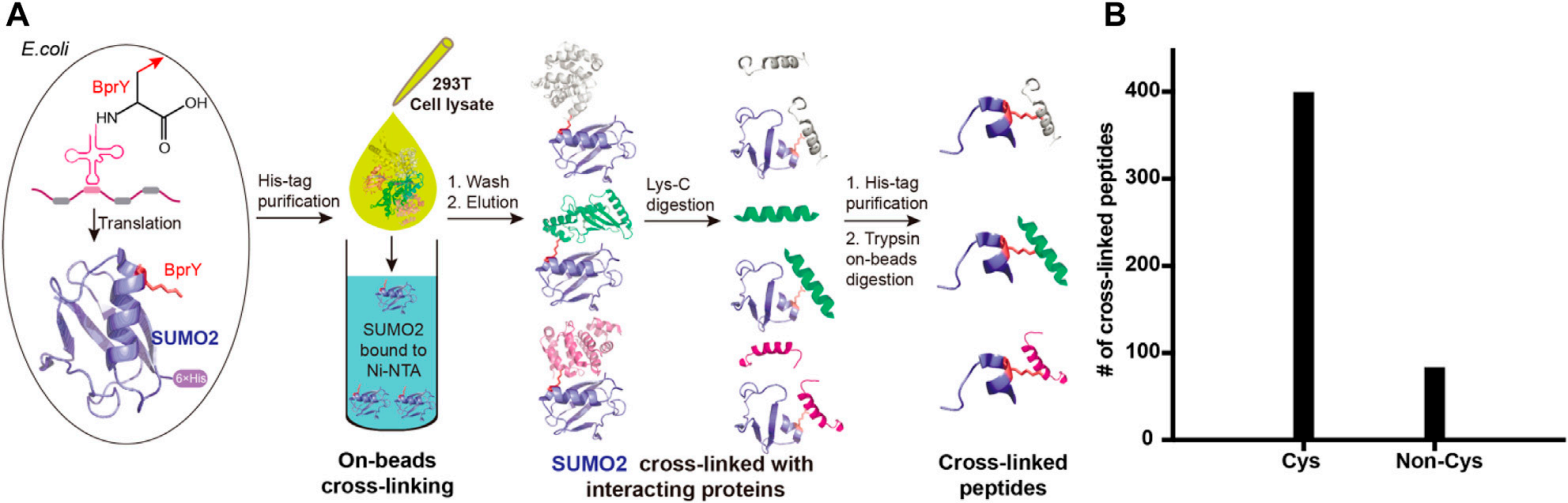
Incorporation of BprY into SUMO2 to identify SUMO2 binders. **(A)** The experimental workflow. Bead-bound SUMO2 was incubated with 293T cell lysates for cross-linking. A double protease cleavage strategy was used to enrich cross-linked peptides before MS analysis. **(B)** The number of cross-linked peptides with Cys or non-Cys residues at cross-linking sites.

*Proteome-wide identification of SUMO2 interacting proteins by BprY*. We next applied BprY to covalently capture SUMO2 interaction partners at the whole proteome level using 293T cell lysates (Figure 4A). After the expression of C-terminal His-tagged SUMO2*(E49BprY) and SUMO2*(R50BprY) in *E. coli* DH10B cells, both constructs were purified by Ni-NTA resin, and the resin-bound SUMO2 was incubated with 293T cell lysates to capture SUMO2 binders by forming cross-links. The resin was then stringently washed followed by elution to give SUMO2 and SUMO2 cross-linked to its interacting proteins. Because there is no lysine residue after K44 in SUMO2, Lys-C digestion followed by a second-step Ni-NTA purification can further enrich cross-linked peptides (Figure 4A). Final on-bead trypsin digestion was done before mass analysis.

**FIGURE 5:**
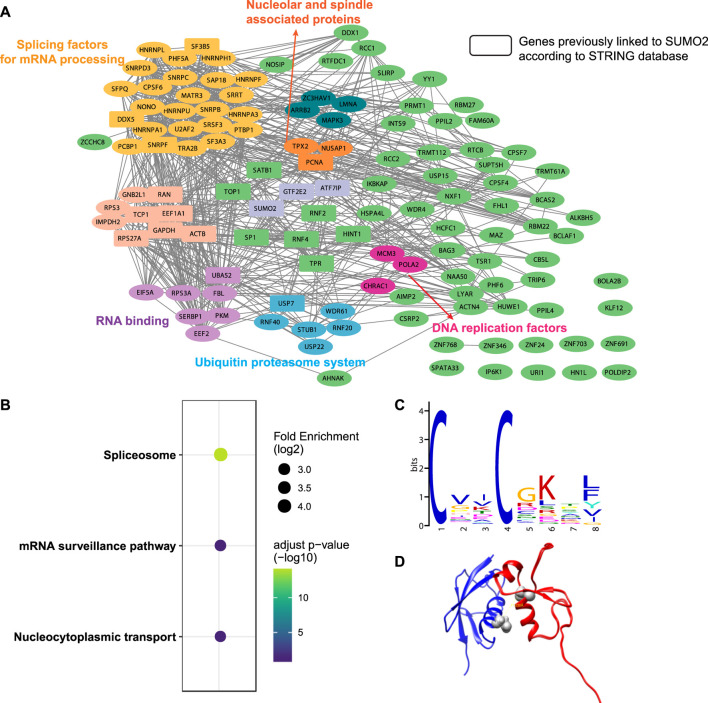
Bioinformatic analysis of SUMO2 interacting proteins. **(A)** STRING analysis identified eight functionally connected clusters of PPIs. Rectangles represent genes previously linked to SUMO2 according to the STRING database. **(B)** KEGG pathways enrichment analysis. **(C)** *De novo* motif discovery by MEME shows a CXXC motif. **(D)** Docking model of SUMO2 (blue, PDB ID 6JXX) and RNF4 zinc finger domain (red, PDB ID 4PPE) using the distance restraint from cross-linking. The cross-linking sites in the SUMO2-RNF4 complex were highlighted in grey.

A total of 482 cross-linked peptides were detected (399 Cys and 83 non-Cys, Figure 4B), corresponding to 264 SUMO2 binders among which 139 are proteins with nucleus localization. Cysteine residues only account for about 2% of the total residues in proteins and likely have low frequency to appear at binding interfaces compared with all the other amino acids investigated here. However, our SUMO interactome data showed that the number of identified cysteine-targeted cross-links is four times higher than that of non-cysteine cross-links. This is mainly due to higher intrinsic reactivity of cysteine sidechain to BprY. We believe that, for non-cysteine cross-links to occur efficiently, the interaction needs to be relatively strong, and the non-cysteine sidechain needs to be in an optimal geometry to react with BprY. Because SUMO2 is mainly localized in the nucleus, we first attempted to analyze these nuclear protein binders. Among them, we observed a previously reported SUMO2 binder RNF4 as well as many known SUMOylation substrates, including PCNA, SATB1, *etc* (Figure 5A). An analysis using the Search Tool for the Retrieval of Interacting Genes/Proteins (STRING) database showed that most of the SUMO2 binders were situated in a large network in which proteins are functionally/physically connected. At medium STRING confidence, 126 of the 139 proteins are in a single interaction network, with a PPI enrichment *p*-value < 1e-16, suggesting the identified SUMO2 binders are functional connected. Furthermore, we performed MCODE analysis on the SUMO2 binder network and identified 8 highly interconnected clusters within the core network (Figure 5A), including the spliceosome, ubiquitin proteasome system, DNA replication factors, and RNA processing factors. Interestingly, members (MCM3/CHRAC1/POLA2) of a cluster are all newly identified SUMO2 binders (Li et al., 2018; Dang and Morales, 2020). Since SUMOylation plays critical roles in DNA damage response (DDR) and numerous SUMO conjugates have been identified, the interaction between SUMO2 and MCM3/CHRAC1/POLA2 may establish a new link between SUMOylation mediated double strand breaks repair and maintenance of genome stability.

The KEGG pathway enrichment analysis highlighted three major pathways associated with these binders—spliceosome, mRNA surveillance pathway, and nucleocytoplasmic transport, consistent with some of the major functions of SUMOylation (Figure 5B). Interestingly, *de novo* motif discovery using MEME based on sequences flanking the cross-linking site of SUMO2 binders revealed a CXXC motif (Figure 5C), which is commonly found in zinc finger proteins. Indeed, many identified proteins have zinc finger domains (ZNF24/346/691/703/768 and ZC3HAV1). This finding is consistent with previous reports suggesting that other than SIMs, zinc fingers can also bind SUMOs (Danielsen et al., 2012; Guzzo et al., 2014; Diehl et al., 2016). Besides, the enrichment of lysine in the motif suggests a close distance between cross-linked sites and SUMOylation sites.

One advantage of GECX-MS is the ability to identify cross-linking sites, which can be used to generate distance restraints for structural modeling. We focused on RNF4, a known binder of SUMO2, and attempted to reveal the potential conformation of the SUMO2-RNF4 complex with molecular docking. RNF4 has four SIMs and a zinc finger domain. Previous studies have identified SIM2 and SIM3 as the major contributor to SUMO binding (Kung et al., 2014; Xu et al., 2014). Interestingly, in this study, we found a cross-link between SUMO2 to the zinc finger domain of RNF4, suggesting that this domain may also be involved in SUMO binding. By applying the distance restraint from cross-linking, we performed molecular docking of SUMO2 and RNF4 zinc finger domain using the Rosetta software suite (Leaver-Fay et al., 2011). The docking model (Figure 5D) revealed a binding interface on SUMO2 close to the SIM-binding groove. One thing to be noted is that because the BprY mediated cross-linking in this study was done in cell lysates with mixed cellular compartments, we also identified many non-nuclear proteins as SUMO2 binders. Gene ontology (GO) analysis suggested a potential link between SUMO2 to protein translation and localization (Supplementary Figure S3). Although there have been studies showing this link (Xu et al., 2010a; Xu et al., 2010b; Chen L.-z. et al., 2014), more detailed investigation will be needed in future studies.

## Conclusion

In conclusion, we have demonstrated that the alkyl bromide containing ncAA BprY can react with not only cysteine residues but also a broad range of nucleophilic amino acids. Therefore, the application of BprY will not be limited to PPIs containing cysteine residues at the binding interface. Indeed, this aspect has been successfully demonstrated by our *in vitro* study of SUMO2/RNF111 interaction in which there are no cysteine residues at the binding site. With this broad reactivity, we applied BprY to covalently capture and identify 264 SUMO2 interacting proteins at a whole proteome level. This study further demonstrated that BprY and the relevant alkyl halide ncAAs are excellent tools to study protein-protein interactions.

## Data Availability

The mass spectrometry proteomics data have been deposited to the ProteomeXchange Consortium *via* the PRIDE (Perez-Riverol et al., 2022) partner repository with the dataset identifier PXD031159 and 10.6019/PXD031159.
