# Supplementary material for: Uncover New Reactivity of Genetically Encoded Alkyl Bromide Non-Canonical Amino Acids
Source: Front Chem. 2022 Feb 18;10:815991. doi: 10.3389/fchem.2022.815991 (PMC8894327; doi:10.3389/fchem.2022.815991)
Supplement: Supplementary file 2 [file DataSheet1.pdf]

Supporting Information

Uncover New Reactivity of Genetically Encoded Alkyl Bromide Non-Canonical Amino Acids

Xin Shu[1, 2†], Sana Asghar[1, 2†], Fan Yang[3], Shang-Tong Li[4*], Haifan Wu[5*], Bing Yang[1,2*]

[1]Zhejiang Provincial Key Laboratory for Cancer Molecular Cell Biology, Life Sciences Institute, Zhejiang University, Hangzhou, Zhejiang, China.

[2]Cancer Center, Zhejiang University, Hangzhou, Zhejiang, China.

[3]Department of Biophysics, Kidney Disease Center of the First Affiliated Hospital, Zhejiang University School of Medicine, Hangzhou, Zhejiang, China.

[4]Glbizzia Biosciences Co., Ltd, Beijing, China

[5]Department of Chemistry and Biochemistry, Wichita State University, Wichita, Kansas, United States.

***Correspondence:**

Shang-Tong Li - Glbizzia Biosciences Co., Ltd; Email: Shangtong.li@glbizzia.com

Haifan Wu - Wichita State University, Wichita, United States; Email: haifan.wu@wichita.edu

Bing Yang - Zhejiang University, Hangzhou, China; Email: bingyang@zju.edu.cn

[†] These authors have contributed equally to this work

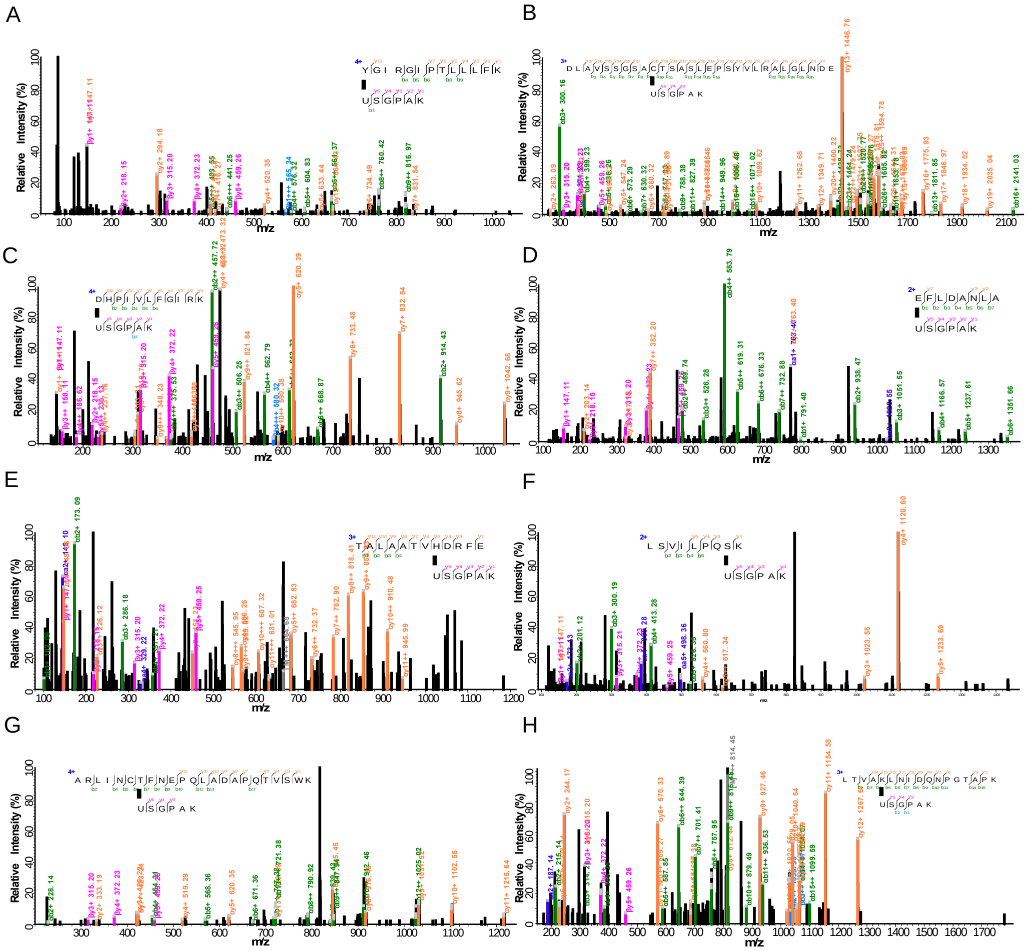

Figure S1. Representative MS/MS spectra of cross-linked peptides from the thioredoxin (Trx) interactome study probed by BprY.

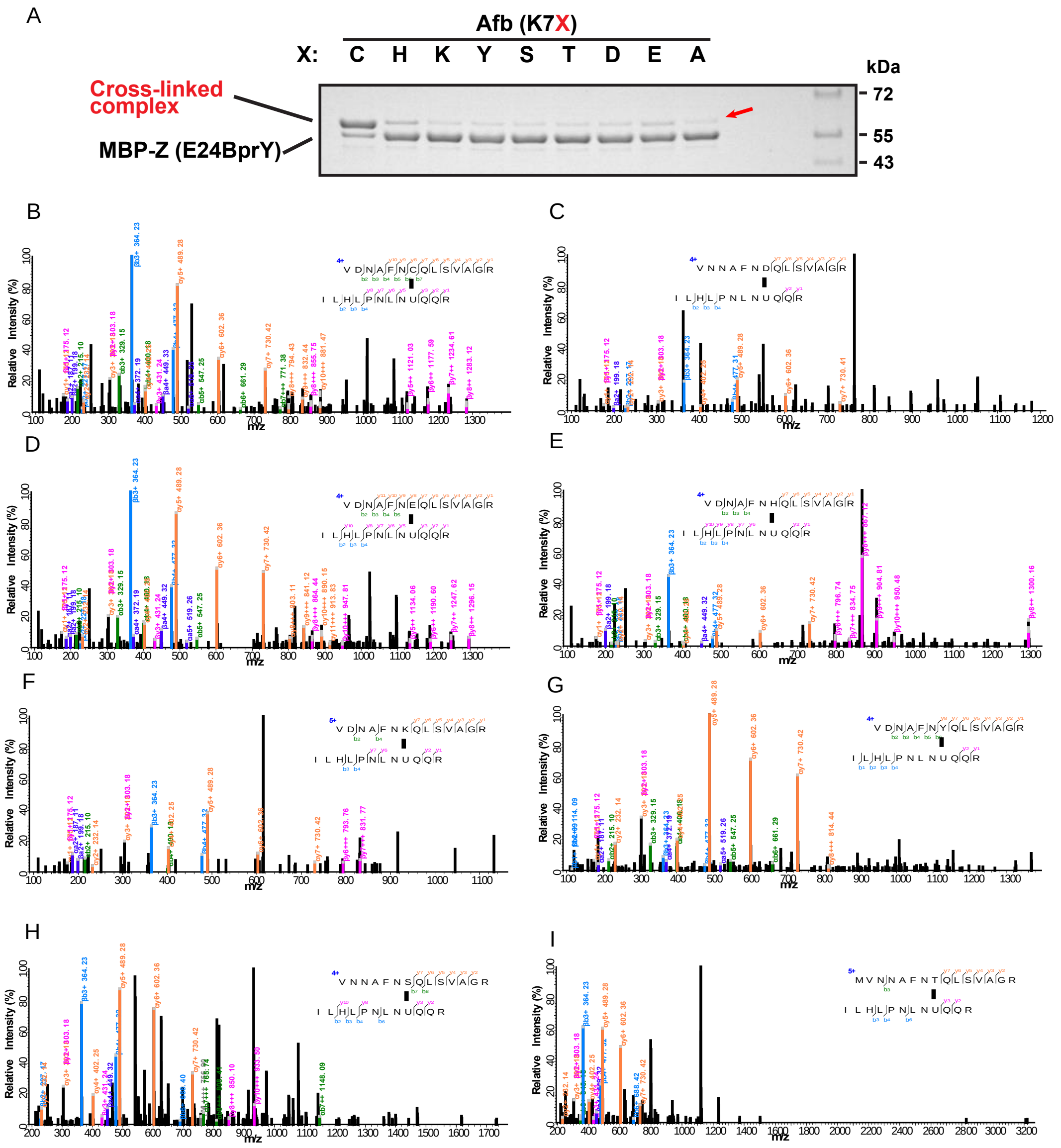

Figure S2. (A) SDS-PAGE gel showing cross-linking between MBP-Z (E24BprY) and Afb (K7X). Red arrow indicates cross-linked bands. (B-I) Representative MS/MS spectra of cross-linked peptides.

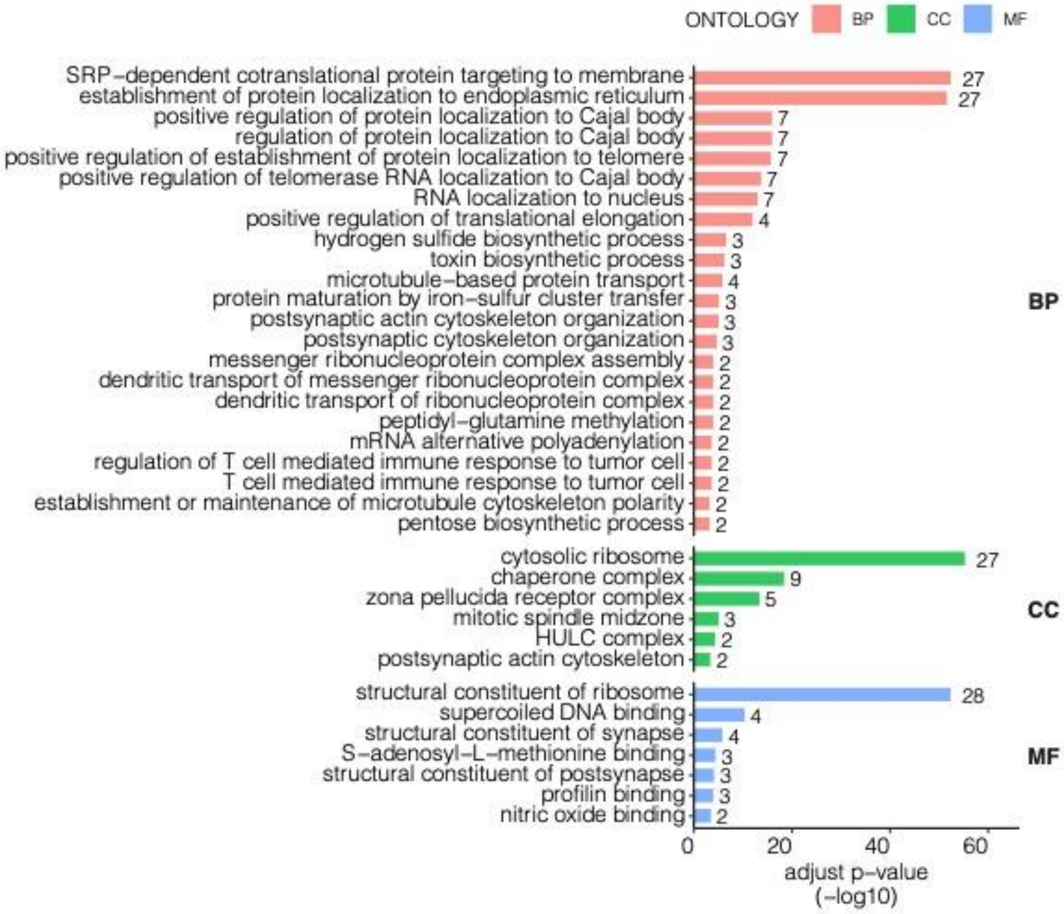

Figure S3. Gene ontology (GO) analysis of all 264 SUMO2 binders.
